# Regional Differences in Sympathetic Nerve Activity Are Generated by Multiple Arterial Baroreflex Loops Arranged in Parallel

**DOI:** 10.3389/fphys.2022.858654

**Published:** 2022-04-04

**Authors:** Kenju Miki, Shizuka Ikegame, Misa Yoshimoto

**Affiliations:** Autonomic Physiology Laboratory, Faculty of Life Science and Human Technology, Nara Women’s University, Kita-Uoya Nishimachi, Nara, Japan

**Keywords:** sympathetic nerve activity, baroreflex, freezing, REM sleep, exercise

## Abstract

In this review, by evaluating the responses during freezing, rapid eye movement (REM) sleep, and treadmill exercise, we discuss how multiple baroreflex loops arranged in parallel act on different organs to modulate sympathetic nerve activity (SNA) in a region-specific and coordinated manner throughout the body. During freezing behaviors, arterial pressure (AP) remains unchanged, heart rate (HR) persistently decreases, renal SNA (RSNA) increases, and lumbar SNA (LSNA) remains unchanged. The baroreflex curve for RSNA shifts upward; that for LSNA remains unchanged; and that for HR shifts to the left. These region-specific changes in baroreflex curves are responsible for the region-specific changes in RSNA, LSNA, and HR during freezing. The decreased HR could allow the heart to conserve energy, which is offset by the increased RSNA caused by decreased vascular conductance, resulting in an unchanged AP. In contrast, the unchanged LSNA leaves the muscles in readiness for fight or flight. During REM sleep, AP increases, RSNA and HR decrease, while LSNA is elevated. The baroreflex curve for RSNA during REM sleep is vertically compressed in comparison with that during non-REM sleep. Cerebral blood flow is elevated while cardiac output is decreased during REM sleep. To address this situation, the brain activates the LSNA selectively, causing muscle vasoconstriction, which overcomes vasodilation of the kidneys as a result of the decreased RSNA and cardiac output. Accordingly, AP can be maintained during REM sleep. During treadmill exercise, AP, HR, and RSNA increase simultaneously. The baroreflex curve for RSNA shifts right-upward with the increased feedback gain, allowing maintenance of a stable AP with significant fluctuations in the vascular conductance of working muscles. Thus, the central nervous system may employ behavior-specific scenarios for modulating baroreflex loops for differential control of SNA, changing the SNA in a region-specific and coordinated manner, and then optimizing circulatory regulation corresponding to different behaviors.

## Introduction

The body is a system of many organs, and each of these organs performs its independent function while coordinating with other organs to achieve an integrated function as a whole body. The parallel arrangement of each organ to the aorta has significant implications: this allows organs to regulate their blood flow independently without being affected by other organs. However, while each organ performs its metabolic activities with its specific organ blood flow, the organs cooperate to maintain an optimal perfusion pressure for organs, namely the arterial pressure (AP), under any change in the physiological states. Sympathetic nerve activity (SNA) plays a primary role in regulating organ blood flow. This review aims to discuss how SNA differently and cooperatively alters the blood flow to each organ and creates the optimal AP for the entire body. In this regard, recent studies have revealed the following three aspects of SNA regulation.

### Multiple Arterial Baroreflex Loops Arranged in Parallel

Multiple arterial baroreflex loops are likely arranged in parallel for each organ. Sagawa proposed the theoretical framework for the parallel arrangement of arterial baroreflex loops and their involvement in AP regulation (Sagawa, 2011). However, there has been a lack of direct evidence supporting this hypothesis. Meanwhile, fragmentary evidence has been reported in anesthetized animals that the neuronal nuclei comprising the baroreceptor reflex pathway respond in a regionally different manner to drug administration. This suggests that the baroreflex pathway has discrete routes for distinct organs and that these pathways act on organs individually and in parallel. It is, therefore, reasonable to assume that the arterial baroreflex system has multiple individually discrete loops, which are arranged in parallel in an organ-specific manner. To experimentally examine this hypothesis, we have generated a full range of arterial baroreflex curves in conscious rats under various conditions (Miki et al., 2003; Nagura et al., 2004; Miki and Yoshimoto, 2018; Kondo et al., 2021).

### Acute Shifts in Arterial Baroreflex Curves

The acute shifts in the input-output relationship of the baroreflex have been consistently induced by changes in daily behaviors such as sleep, exercise, mental stress (Miki and Yoshimoto, 2005), and pathological conditions such as heart failure (Xing et al., 2014). Input-out data of the arterial baroreflex has generally been fitted to a sigmoid curve to assess the shifts in the baroreflex loop. Kent’s sigmoid curve has been widely used for this purpose (Kent et al., 1972). This model is used to quantify the feedback gain of the arterial baroreflex and changes in the maximum capacity of feedback regulation, AP threshold, etc. (Potts et al., 1993; Nagura et al., 2004); the changes in these variables have served as the basis for the discussion of the involvement of the central nervous system (CNS) above the medulla in the shift.

### Regional Differences in Sympathetic Nerve Activity

Regional differences in SNA have been observed in many species, including rats, rabbits, sheep, and humans where experiments were carried out either in anesthetized or conscious states (Morrison, 2001; Park et al., 2008; Yoshimoto et al., 2011; Xing et al., 2014; Kondo et al., 2021). While there are many interesting issues to address regarding methods for measuring SNA (Malpas, 2010; Stocker and Muntzel, 2013; Hart et al., 2017), this review will focus primarily on the regional differences in SNA measured simultaneously at multiple sites in the same preparation. To assess regional differences in SNA, simultaneous measurement of the SNA in multiple regions in the same preparation is essential. Considering the difficulty in evaluating the absolute value of SNA, comparisons of changes in SNA by region should be made only when there is a clear difference between SNAs. However, comparing the degree of change in SNAs measured in different animals is challenging. Moreover, anesthesia has a profound effect on SNA (Stock et al., 1978; DeLalio and Stocker, 2021). Suppression of the higher CNS by anesthesia may affect the response of regional differences in SNA. Therefore, simultaneous measurement of SNA at multiple sites in conscious animals may have some advantages in studying the mechanism of regional differences in SNA.

In this review, we are discussing the functional diversity of the RSNA and LSNA, but what we are measuring is the extracellular potential of sympathetic fiber bundles, which also have even more diverse functions. For instance, RSNA is a recording of the extracellular potential of a mixture of the sympathetic fibers; it mediate vasoconstriction, Na^+^ reabsorption, and renin release (DiBona and Kopp, 1997). The diverse functions of RNSA and LSNA itself need to be recognized, but this issue is out of the scope of the present review.

### Central Scenarios Modulating Baroreflex Loops and Sympathetic Nerve Activity in a Region-specific and Coordinated Manner

To summarize the above three points, the arterial baroreflex has multiple pathways, and these are arranged in parallel to change the SNA in a regionally different manner, creating region-specific changes in the SNA of organs. We have been evaluating the arterial baroreflex by simultaneously and continuously measuring renal SNA (RSNA), lumbar SNA (LSNA), HR, and AP in conscious rats (Miki et al., 2003; Nagura et al., 2004; Kondo et al., 2021). We found that RSNA, LSNA, and HR changed in the same way in some cases, and in other cases, they changed in a region-specific manner. These data indicate that the CNS can modify SNA in an essentially region-specific manner and that these changes are coordinated and strategic in regulating AP. The following three examples, which pertain to freezing, rapid eye movement (REM) sleep, and treadmill exercise, illustrate how the arterial baroreflex loop can alter SNA in a region-specific, coordinated, and strategic manner. These examples highlight sophisticated control scenarios operated through arterial baroreflex loops to optimally regulate AP under different physiological situations.

## Freezing: Innate Strategies for Increasing the Chance of Survival Against Threats and Potential Danger

The freezing behavior is one of the innate responses of animals and can be classified as a preparatory reflex for threats and potential danger (Dampney, 2015; Kozlowska et al., 2015). When encountering unmanageable stresses, including predators, animals will freeze to conserve energy and create a state of readiness for fight or flight, thereby increasing the probability of survival. In humans, when exposed to unmanageable stresses such as natural disasters, individuals become apathetic and exhibit behaviors like freezing (Hagenaars et al., 2014). During freezing behavior, SNA plays a dominant role in modulating cardiovascular functions strategically to make the entire body ready for fight or flight.

**FIGURE 1:**
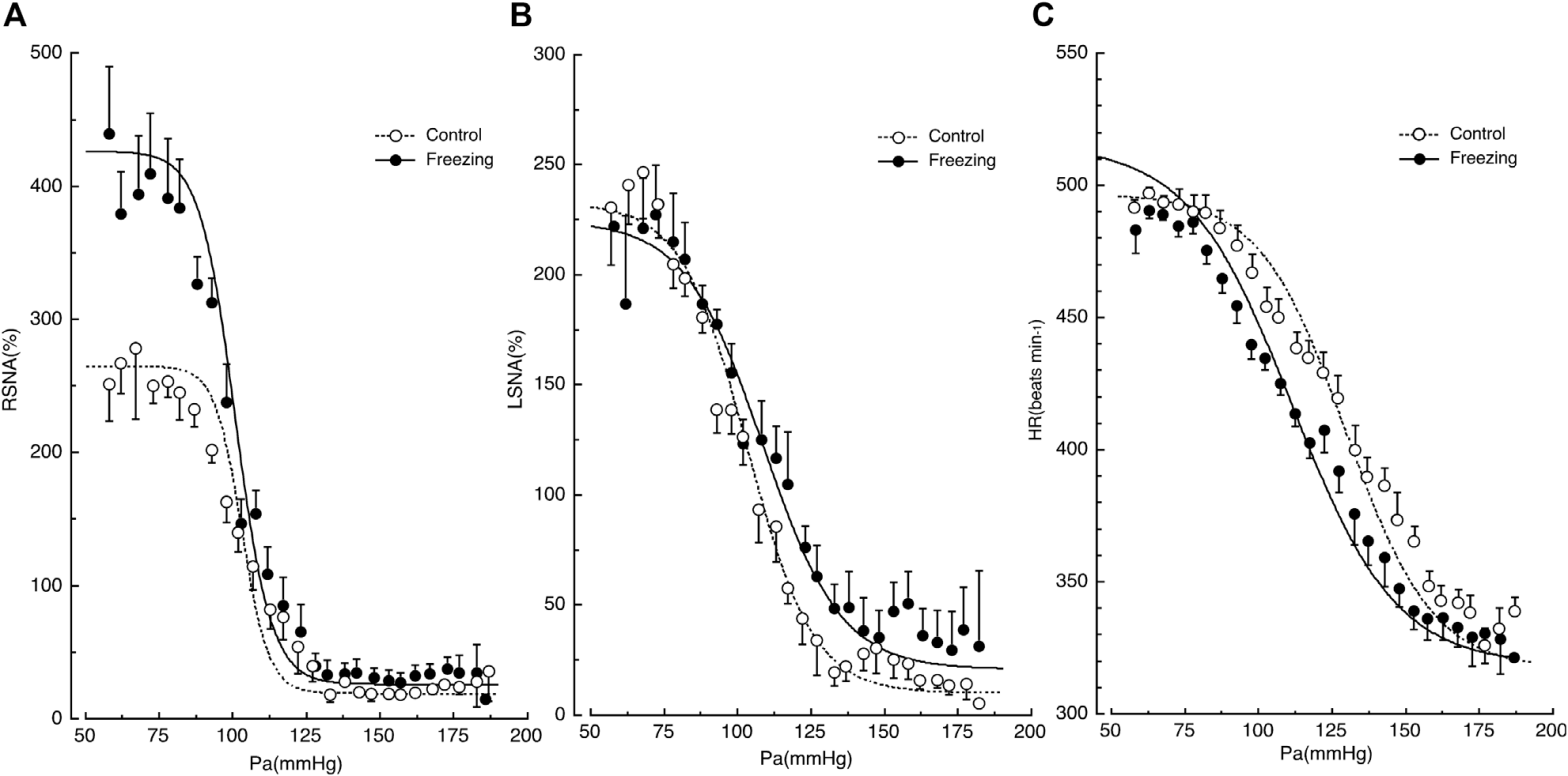
Shifts in the baroreflex curves for renal sympathetic nerve activity (RSNA) in panel **(A)**, lumbar sympathetic nerve activity (LSNA) in panel **(B)**, and heart rate (HR) in panel **(C)** during the control and freezing periods. AP, arterial pressure. Symbols and bars indicate the mean and standard error of mean, respectively. The image was obtained with permission from the study by Kondo et al. (2021).

When rats are exposed to loud noise, they exhibit freezing behavior in which the AP remains the same while HR decreases persistently, RSNA increases, and LSNA remains unchanged (Yoshimoto et al., 2010). The baroreflex curves for RSNA, LSNA, and HR are shifted in a region-specific manner during freezing behavior, as shown in Figure 1 (Kondo et al., 2021). The rats remained still during freezing because they showed no change in muscle activity, which allows us to generate an entire range of baroreflex curves by administrating vasoactive drugs, because without any other disturbance, including spontaneous moving (Kondo et al., 2021). In conscious rats, artificial changes in AP sometimes produce spontaneous movements, which may alter SNA and HR. Therefore, it is not likely that muscle ergoreceptors would cause an increase in RSNA, as seen during exercise (Fisher et al., 2015). Consequently, it is highly possible that the region-specific changes in RSNA, LSNA, HR, and AP observed during freezing behavior are attributable to the survival strategy scenario in the CNS. The different shifts in the baroreflex curves for RSNA, LSNA, and HR are elicited by CNS, including the defense area (Coote et al., 1979; Hilton, 1982; Kozlowska et al., 2015) and the medullary baroreflex pathway (Dampney, 2015).

### Region-specific Shifts in Baroreflex Curves

First, the baroreflex curve for RSNA shifted upward with a significant increase of 150% in the upper plateau and with no changes in the lower plateau (Kondo et al., 2021), while RSNA increased significantly during freezing. Thus, the CNS increases the maximal drive of RSNA to the kidney by 1.5-fold during freezing behavior, potentiating the baroreflex ability.

Second, the baroreflex curve for LSNA remained the same in comparison with the control state (Kondo et al., 2021), while LSNA did not change significantly during freezing. This suggests that the SNA drive to the muscle is not altered during freezing behavior over the entire range of AP changes. LSNA shows a negative correlation with muscle blood flow (Miki et al., 2004). Since freezing behavior is a state of readiness for fight or flight, the CNS may encounter a scenario where increased LSNA and suppressed muscle blood flow are not desirable in the same way that the increased RSNA suppressed renal blood flow.

Third, the baroreflex curve for HR shifted leftward during freezing behavior in a parallel manner, with significant reductions in the midpoint and threshold pressure (Kondo et al., 2021). This implies that the HR is set to lower levels across the entire range of AP changes. The reduction in HR offsets the reductions in renal vascular conductance due to the increase in RSNA, resulting in no change in AP during the freeze behavior. Furthermore, the decrease in HR allows myocardial energy to be conserved for the transition from freezing to the fight-or-flight state, when the heart’s performance is rapidly activated.

These region-specific changes in baroreflex curves seem to represent a highly sophisticated strategy for survival. The heart conserves energy by decreasing cardiac function, and the resulting reduction in cardiac output is offset by a reduction in vascular conductance due to increased SNA in the kidneys and possibly the visceral system, leaving AP unchanged. Simultaneously, the LSNA remains unchanged, and muscles can be left in a state of readiness to be active at any time.

In summary, the CNS can modulate the baroreflex curves in a region-specific manner, resulting in regionally variable changes in SNA and causing changes in the function of each organ independently, while they are coordinately operated for survival during freezing behavior.

## Rapid Eye Movement Sleep: A Paradox of Opposite Directional Changes in Sympathetic Nerve Activity

Sleep is classified primarily into non-rapid eye movement (NREM) sleep and REM sleep. During NREM sleep, the RSNA, LSNA, and HR are all decreased, and the AP is low and stable. REM sleep is also known as the autonomic storm period and paradoxical sleep. During REM sleep, AP rises and becomes unstable. RSNA decreases while LSNA increases during REM sleep, thus undergoing opposite changes. SNA and AP regulation during REM is unique and paradoxical compared to that in other physiological states, as shown in Figure 3 (Nagura et al., 2004; Yoshimoto et al., 2011). The possible reasons why SNA and AP regulation during REM sleep are unique are discussed below.

### Vertical Suppression of the Baroreflex Curve for Renal Sympathetic Nerve Activity During Rapid Eye Movement Sleep

We reported that AP increased by 5 mmHg, and RSNA decreased by 45% during REM sleep (Nagura et al., 2004). While this might imply that the reduction in RSNA was caused by simple baroreflex, the reduction is more likely attributable to the change in the baroreflex curve for RSNA, which is vertically suppressed during REM sleep in comparison with NREM sleep. Vertical compression is characterized by a significant decrease in the maximum level (upper plateau) and maximum gain (Nagura et al., 2004). This shift in the baroreflex curve causes a reduction in RSNA even though the AP does not change. A similar shift in the baroreflex curve for RSNA has been observed after treadmill exercise when the RSNA decreases by 25% even with no change in AP (Miki et al., 2003). Thus, the CNS actively reduces RSNA during REM sleep, thereby increasing the vascular conductance of the kidneys (Yoshimoto et al., 2004).

**FIGURE 2:**
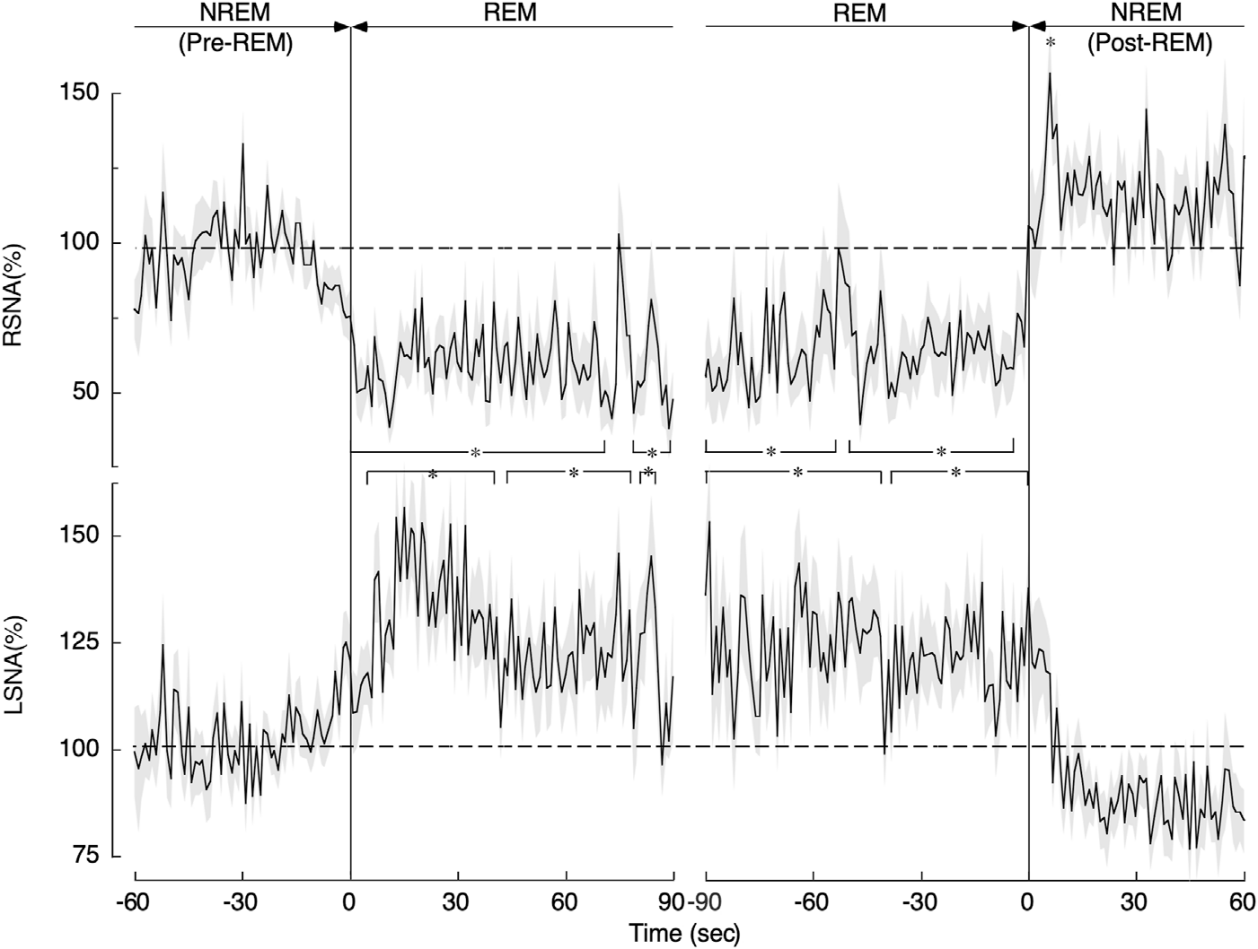
Changes in renal sympathetic nerve activity (RSNA) and lumbar sympathetic nerve activity (LSNA) during the transition from non-rapid eye movement (NREM) sleep (pre-REM) to REM sleep and from the REM sleep to NREM (post-REM) sleep periods. Continuous drawn lines represent mean values and shaded areas above and below show the standard error of mean. *p < 0.05 indicates a significant difference from the average level obtained during the pre-REM period. The image was obtained with permission from the study by Yoshimoto et al. (2011).

In contrast to the reduction in RSNA, LSNA increased significantly in a stepwise manner during REM sleep, as shown in Figure 2. Unfortunately, the changes in the baroreflex curve for LSNA have not been reported yet. Since LSNA increased in a sustained manner during REM sleep, we may speculate that the baroreflex curve for LSNA somehow likely shifted rightward. Muscle atonia occurs during REM sleep, thereby reducing the vascular conductance of muscles throughout the body. Moreover, the increased LSNA, which mainly innervates the muscles of the lower extremities, decreases the vascular conductance of the muscles of the lower extremities.

### Physiological Contexts for the Reversed Sympathetic Nerve Activity Changes Occurring in Rapid Eye Movement Sleep

It is interesting to speculate why RSNA and LSNA change in opposite directions during REM sleep and why the vascular conductance of the organs they also innervate changes in different directions. REM sleep is a unique state in which brain activity increases to the level of awake states. The brain absorbs a large amount of the cardiac output; total cerebral blood flow at rest is 15–20% of total cardiac output in humans (Folino, 2006). To meet the increased metabolic demand of the brain during REM sleep, blood flow to the brain must increase. Besides the increase in brain blood flow, blood flow to the kidneys and splanchnic organs is also increased (Miki et al., 2004). Paradoxically, it is important to note that cardiac performance was the lowest during REM sleep (Miller and Horvath, 1976). The cardiac output changes with muscle activity but does not change in parallel with that of the brain. However, the brain can control SNA and the baroreflex curves. The brain activates muscle SNA selectively, causing vasoconstriction of the muscle, which is the largest organ in the body. This, in turn, overcomes the vasodilatation of the kidneys, splanchnic organs, and brain, as well as the reduction in cardiac output, such that AP can be maintained during REM sleep.

## Exercise: Simultaneous Increases in Sympathetic Nerve Activity

Exercise involves muscle contraction, which causes muscle metabolism and requires increased muscle blood flow (Rowell, 1986). Meeting the muscle blood flow requirements to increase the metabolic rate is crucial for the performance of the exercise. SNA plays an essential role in adjusting muscle blood flow during exercise. The exercise-induced increase in SNA acts on the capacitance vessels, myocardial contractility, and cardiac pacemaker, increasing cardiac output to increase to nearly five times the resting level. In addition, increased SNA causes vasoconstriction of resistance vessels in inactive muscles and visceral organs, compensating for the increase in vascular conductance caused by muscle contraction. As a result, AP increases during exercise. Maintenance of high AP during exercise is vital to ensure stable muscle blood flow when large-volume muscles contract and vasodilation occurs. Furthermore, a striking variation in central venous pressure has been observed during dynamic exercise due to the mechanical effect of the muscle pump (Rowell, 1986). Since this leads to fluctuations in cardiac output, maintaining an adequately high AP is crucial to keep muscle blood flow during exercise.

**FIGURE 3:**
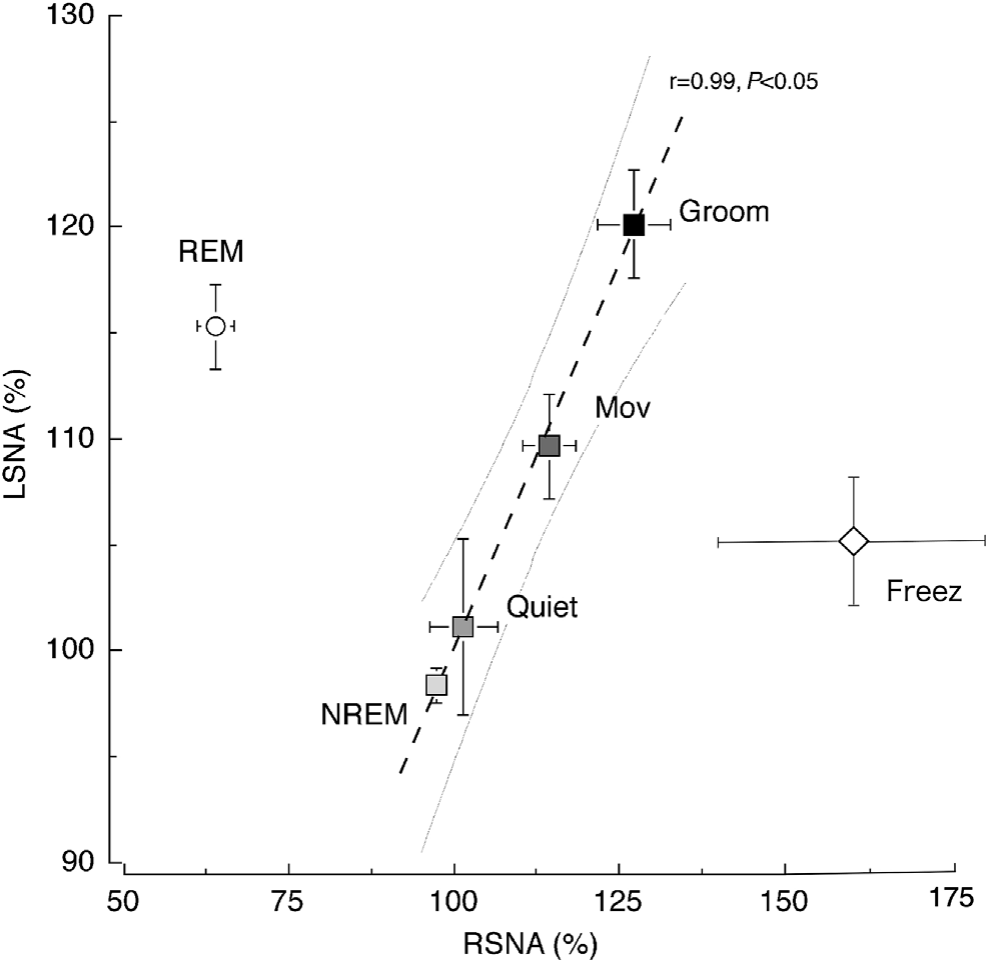
Relationship between renal sympathetic nerve activity (RSNA) and lumbar sympathetic nerve activity (LSNA) across the behavioral states. A significant (*p* < 0.05) linear relationship was observed between RSNA and LSNA (y = 0.72x + 27.76) in the non-rapid eye movement (NREM) sleep, quiet awake (Quiet), moving (Mov), and grooming (Groom) states. The relationship between RSNA and LSNA during the REM sleep and freezing period (Freez) was dissociated from the line obtained during other behavioral states. Values are shown as the mean ± standard error of the mean. The gray curved lines are the 95% confidence bands for the regression line. The image was obtained with permission from the studies by Yoshimoto et al. (2010; 2011).

SNA is believed to increase throughout the body during exercise. Figure 3 shows the relationship between RSNA and LSNA with increasing exercise intensity in daily activity (Yoshimoto et al., 2011). The RSNA and LSNA increase linearly from NREM sleep to the grooming state, except during REM sleep. In humans, muscle SNA is slightly suppressed by light exercise but increases linearly with exercise intensity (Saito et al., 1993). There is substantial evidence for intensity and duration-dependent increases in muscle SNA to both the active and inactive limbs during isometric and dynamic exercise in humans (Fisher et al., 2015). SNA is likely to increase in most organs in proportion to the magnitude of the increase in muscle activity.

### Exercise Shifts the Baroreflex Curve for Renal Sympathetic Nerve Activity Upward to the Right Compared to the Resting State

**FIGURE 4:**
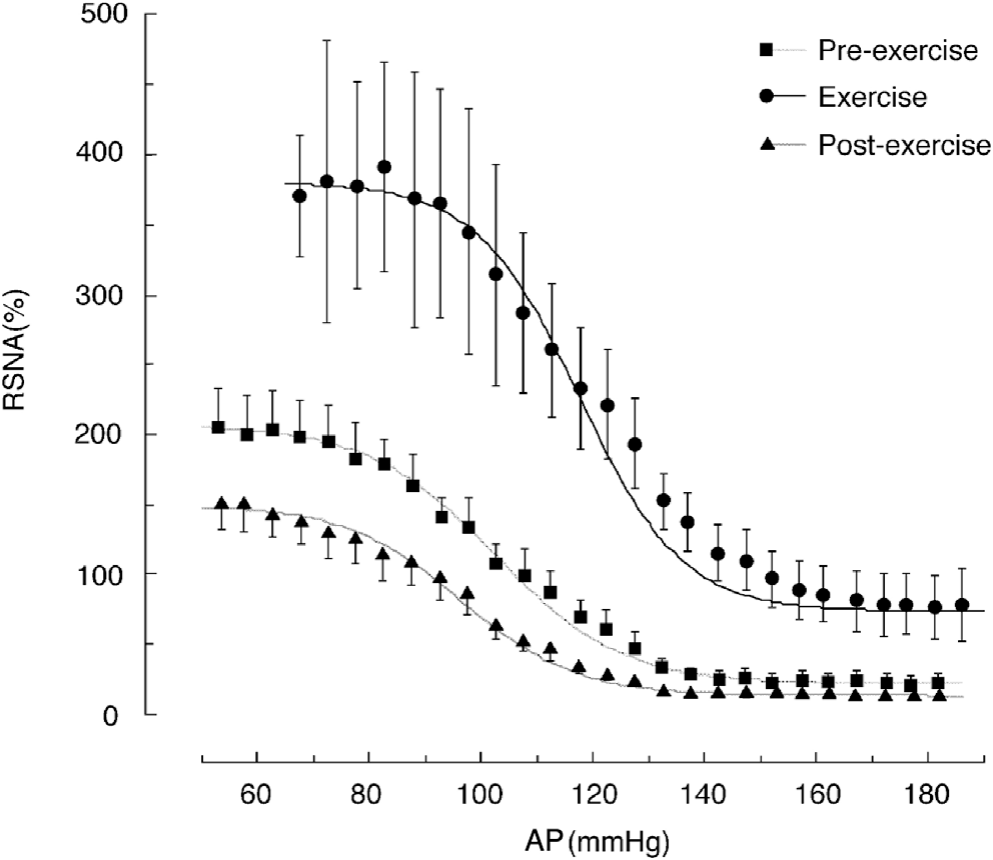
Shifts in the baroreflex curves for renal sympathetic nerve activity (RSNA) during the pre-exercise, treadmill exercise, and post-exercise periods. The average oxygen consumption during treadmill exercise at a speed of 20 m/min with a 0% gradient performed in this study was 51 ml/min/kg, which was approximately 70% of the maximum oxygen consumption. The curves and bars indicate the mean and standard error of the mean, respectively, estimated over each 2.5-mmHg bin of arterial pressure (AP). The image was obtained with permission from the study by Miki et al. (2003).

Figure 4 shows the shifts in the baroreflex curve for RSNA before, during, after the treadmill exercise (about 70% VO_2_Max) in rats (Miki et al., 2003). Full-range baroreflex curves were generated by intravenously administering phenylephrine and nitroprusside. This right-upward shift in the baroreflex curve for RSNA allows AP and RSNA to increase simultaneously during exercise and increases the feedback gain to enhance the ability to suppress AP fluctuations. The shifts in the baroreflex curve for RSNA can be characterized into three parts: 1) an increase in central AP (rightward shift), 2) an increase in the minimum response level (upward shift), and 3) an increase in the feedback gain caused by the increase in response range of RSNA. For the mechanism underlying the shift in the baroreflex curve, please refer to the excellent review by Dampney (Dampney, 2017). Here, the significance of these three points in the regulation of circulation during exercise is addressed.

First, the rightward shift of the baroreflex curve for RSNA is indicated by an increase in the central blood pressure of the curve. The proximity of the prevailing AP during exercise to the central pressure is of significance. Thus, the AP during exercise is near the highest value of the feedback gain, providing the output of SNA at a high gain. In addition, it can respond to SNA in the same way even if it decreases or increases from the mean AP value.

Second, the upward shift of the baroreflex curve for RSNA is attributed to a significant increase in the minimum response level of SNA. At rest, when the AP rises, SNA is suppressed and reaches almost the zero level, and the contractile effect of SNA on peripheral blood vessels ceases. However, during exercise, even if the AP rises to a high level, SNA maintains a certain level of activity and can continuously maintain vasoconstriction of peripheral organs. This minimal level of SNA causes the resistance vessels of inactive muscles and internal organs throughout the body to constrict, thereby supporting increased blood flow to the active muscles.

Third, the feedback gain is the first derivative of the baroreflex curve and shows its maximum value at the central blood pressure value of the sigmoid curve. The gain curve of RSNA shifts to the right (hypertensive side) during treadmill exercise in rats, and the peak value increases by 2-fold (Miki et al., 2003). Thus, the degree of response of SNA is higher for the same AP variation, indicating that changes in SNA increase the ability to suppress AP fluctuations through feedback regulation, allowing for stable AP control during exercise.

By contrast, exercise shifts the baroreflex curve for HR as well (Ogoh et al., 2005; Raven et al., 2006), which is different from the shift of the baroreflex curve for SNA as described above. This difference may be due to regional differences in cardiac SNA, involvement of cardiac vagal nerve activity, and maximum heart rate. In particular, the changes in cardiac vagal activity during exercise have not been measured and are not known at present (Gourine and Ackland, 2019). Shifts in the baroreflex curve for HR and variability of HR are sometimes used for estimating changes in SNA (Monahan, 2007; Billman, 2013; Marmerstein et al., 2021). However, the baroreflex curves for HR and SNA are fundamentally different reflex loops, thus caution should be taken when making such extrapolations.

In summary, SNA increases uniformly throughout the body during exercise. The baroreflex curve for RSNA shifts right-upward, and a similar shift is thought to occur in the other SNA baroreflex curves. As a result, AP and SNA increase simultaneously during exercise. Besides, exercise increases the feedback gain, making it possible to maintain stable AP with a significant fluctuation in the vascular conductance of working muscles.

## Summary and Perspective

SNA shows regional differences in response to behavior and external stress, and these differences are accompanied by region-specific and behavior-specific shifts in the baroreflex curves for SNA. This review attempted to elucidate the reasons underlying the region-specific shifts in arterial baroreflex loops and their implications, focusing on three examples: freezing, REM sleep, and treadmill exercise. The shifts in the arterial baroreflex loop are highly plausible from the viewpoint of integrating AP regulation. Notably, the baroreflex loop acts independently and in parallel on each organ. These parallel arterial baroreflex loops account for regional differences in SNA. The CNS above the medulla can modulate each parallel baroreflex curve separately, producing context-specific changes in SNA: to prepare for battle against an inexorable enemy and increase the chances of survival during the freeze; to maintain cerebral blood flow during REM; and to maintain stable muscle blood flow during exercise. The CNS above the medulla is exposed to a number of scenarios that would change the baroreflex curve in a region-specific manner, and clarification of scenarios in contexts other than those discussed here is awaited.

**FIGURE 5:**
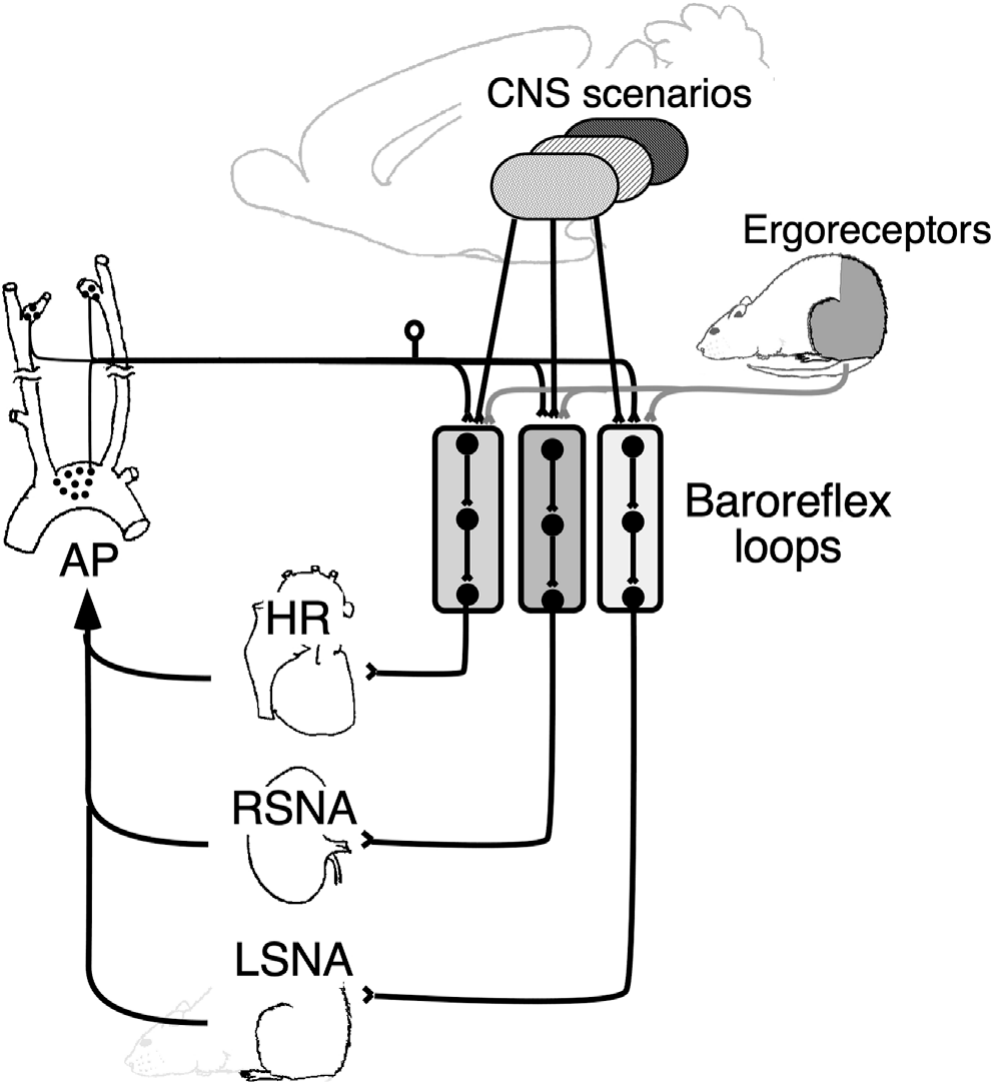
Schematic diagram showing the role of parallel baroreflex loops in regulating sympathetic nerve activity and, thereby, arterial pressure (AP). Parallel baroreflex loops regulate heart rate (HR), renal sympathetic nerve activity (RSNA), and lumbar sympathetic nerve activity (LSNA) separately to generate region-specific changes in the SNA. Each arterial baroreflex loop receives similar stimulus information from the muscle ergoreceptors, but it receives separate and integrated information from the CNS above the medulla to optimize AP as a whole body.

Figure 5 shows a conceptual diagram of a multiple parallel arterial baroreflex loop. This view is consistent with previous studies conducted under anesthesia, showing discrete conduits within the central arterial baroreflex pathway, including the nucleus tractus solitarius (Polson et al., 2007; Scislo et al., 2008), caudal ventrolateral medulla (Miyawaki et al., 1997), and rostral ventrolateral medulla (McAllen and May 1994; Mueller et al., 2011; Farmer et al., 2019). Exactly how these nuclei relate to each other to cause region-specific changes in SNA, or whether they dynamically alter their networks, is of great interest and remains to be studied.

The blood supply of the individual organs is regulated by a balance between the local independent regulatory system, including autoregulation, and the central scenario that determines the optimal distribution of organ blood flow adapting to various physiological states. Multilayered redundancy of regulatory systems may work for keeping optimal organ blood flow adapting to various physiological states. The baroreflex is one of the control systems of central nervous system origin and is one of the components constituting a multilayered and redundant control system. In this review, we discussed shifts in the baroreflex curve in normal conditions, but the shifts may also occur in cardiovascular diseases and, in some cases, may not function properly. Further studies are needed to elucidate the details of the dysfunctional baroreflex curves for SNA and how it interacts with other regulatory systems, including the local autoregulation system in cardiovascular diseases.
